# Children's Hospital, Birmingham

**Published:** 1921-02-12

**Authors:** 


### Children's Hospital, Birmingham.

The report of the Committee of Management for the quarter ended December 31 last contains some interesting facts and figures. The reception of paying patients in the building in Ladywood Road, to which we referred in The Hospital of January 29, is explained. It is a part of the Committee's plan to make the resources of the hospital and its out-patient department available in connection with the Infant Welfare scheme of the city and the medical treatment of school children under the education authorities. It is hoped to provide for the opening of two empty wards for these cases, payment for the maintenance to be defrayed by the local authorities. The *x*-ray department has been completed and opened. The present out-patient department, in Steelhouse Lane, is not satisfactory, and the Committee is anxious to undertake the building of a new out-patient department on the site provided at the time of the erection of the new hospital in Ladywood Road. It recommends that one-half of all free legacies received be allotted to a fund for the completion of the hospital, and more especially the building of the out-patient department. By an alteration in the laws a year ago the Board was empowered to use half the amount of free legacies for the current needs of the hospital should there be an adverse balance on any year's working, and the present recommendation refers to the remaining half. The financial statement for the quarter shows the income to be £10,091 17s. 6d., as against £5,489 17s. 10d. for the corresponding quarter of 1919; in the former figure is included a sum of £2,100 received from the National Relief Fund as a special donation towards the war deficit. Deducting this, the net increase in income is very substantial and noteworthy. The expenditure for the three months was £5,756 14s. 11d., compared with £4,928 in the previous year. The deficit for the year, however, amounts to £5,000, and with £12,961 brought forward from 1919, makes the accumulated deficit to date £17,961. This will be somewhat reduced by applying the power granted to use one-half of the free legacies for current needs.

